# Exploring Barriers to Recovery Amongst Women with Psychosis: A Qualitative Secondary Analysis

**DOI:** 10.1007/s11013-026-09985-0

**Published:** 2026-04-17

**Authors:** Chizara Lock, Anna Lavis, Rosina Pendrous, Sheila Greenfield

**Affiliations:** 1 Department of Applied Health Sciences, University of Birmingham, Birmingham, UK; 2 Institite for Mental Health, University of Birmingham, Birmingham, UK

**Keywords:** Psychosis, Recovery, Anthropology, Women, Gender

## Abstract

Experiencing psychosis can impact all areas of a person's life, causing significant changes to thoughts, perceptions, mood, behaviour, and sense of self. Details of the specific barriers to recovery experienced by women with psychosis and how these barriers may relate to both sex and gender remain unknown. To identify and conceptualise barriers to recovery, a qualitative secondary analysis was undertaken of 31 semi-structured interviews from a primary anthropological study focused on women’s lived experiences of a first episode of psychosis. Participants were recruited from Early Intervention Services in England, UK, between 2010 and 2015. Reflexive thematic analysis demonstrated various barriers to recovery, including internal conflicts with identity, the constraining of moral agency, inadequate support to address past traumas, structural factors, and stigma. Each of these barriers intersects with both sex and gender norms in a number of ways. Barriers to recovery must be addressed within mental health services to ensure that women have the best chance of moving forward with, and finding new meaning in, their lives after psychosis. Consideration of past experiences as well as normative gender roles, and other structural barriers is needed. Future research should develop and evaluate sex- and gender-specific interventions and consider integrating these into clinical practice.

## Introduction

It is known that recovery from psychosis is profoundly shaped by social and cultural contexts. Anthropological and sociological research has shown that illness and recovery are deeply embedded in social worlds, where meanings, relationships, and structures of inequality shape both the course of illness and the possibilities for recovery (Estroff, 1989; Kleinman, 1988). Factors such as socioeconomic background, ethnicity, and interpersonal relationships influence both vulnerability and recovery trajectories (Hoertnagl et al., 2020; Monte et al., 2008).

Psychosis, often defined within biomedical discourse by the presence of hallucinations, delusions or both (National Institute for Health and Care Excellence, 2014), has an estimated England wide prevalence of 0.7% (McManus et al., 2016). It carries a substantial disease burden (GBD 2019 Mental Disorders Collaborators, 2022) and can be functionally debilitating, with significant impact, for example, on relationships and employment (Rinaldi et al., 2010). Additionally, medications that are commonly prescribed to treat psychosis are known to engender unfavourable side effects (Seeman, 2012), thereby adding to the lived impact of psychosis itself.

First Episode Psychosis (FEP) describes the first onset of psychotic symptoms (National Institute for Health and Care Excellence, 2016). The term Duration of Untreated Psychosis (DUP) quantifies the time-period from first onset of symptoms to initiation of treatment and it is recognised that a longer DUP can lead to poorer prognostic outcomes (Marshall et al., 2005). Early Intervention Services (EIS) were introduced to the UK in the 1990s as multi-disciplinary mental health teams that aimed to reduce DUP and promote recovery for people presenting with psychotic symptoms between the ages of 14 and 65 years (Joseph & Birchwood, 2005). Whilst EIS are a cornerstone of the UK’s mental health services, little attention has been paid to how women in particular experience recovery within these services, or how gendered social expectations may shape their outcomes.

Recent studies have recognised sex differences in psychosis, such as age of onset, symptom presentation and responsiveness to treatment, as well as recovery (Ayesa-Arriola et al., 2020; Carter, et al., 2022). Males tend to have an earlier age of onset, with a peak incidence in their early 20’s. Amongst females there are two distinct peaks of incidence: late 20’s and over the age of 40, around menopause (Giordano et al., 2021). This variation may be caused by hormonal differences, such as in oestrogen, which is believed to be neuroprotective (Riecher-Rössler et al., 2018), as well as differences in brain maturation and genetic vulnerabilities (Abel et al., 2010). Importantly, epidemiological research has shown that females tend to have higher recovery rates across cultures (Hopper, 2008), which highlights the importance of considering sex as a variable that shapes not only symptom presentation but also outcomes.

In much existing research on psychosis, sex is defined and classified biologically, but there is also a need to pay attention to gender and its social construction. Encompassing the roles, behaviours, and identities that societies attribute to individuals, gender is influenced and shaped by cultural norms and expectations, and it has long been recognised that these can have substantial effects on health (Mauvais-Jarvis et al., 2020; Verbrugge, 1985). As such, consideration of both gender and sex is required to understand the interplay between biological and socio-cultural factors that may shape women’s experiences of psychosis and recovery.

What recovery means, how it relates to personhood, and how it might be achieved has long been subject to debate that has seen clinical perspectives challenged by those emerging from the expertise of lived experience. Rather than focusing on deficits, disabilities, and a fixed end point for recovery, the recovery movement was forged by survivor researchers and user-led advocacy in the 1980s (Deegan, 1988). This called for reconceptualising recovery as a personal process that involves rebuilding a sense of agency, identity, and connectedness, and not only—or even not at all—the eradication of so-called ‘symptoms’ such as hearing voices (Bellack, 2006; Law & Morrison, 2014; Leamy et al., 2011; Pilgrim & McCranie, 2013; Pitt et al., 2007). This reconceptualisation has shaped the personal recovery model (Douglas et al., 2022) that is increasingly utilised within UK mental health services, including in EIS (Department of Health & Social Care, 2011; Law et al., 2012). 

Specifically, Leamy, et al. (2011) operationalised personal recovery in proposing a conceptual framework based on the findings from a qualitative review of 115 studies; CHIME (Connectedness, Hope, Identity, Meaning, and Empowerment) (Hurst et al., 2022; Piat et al., 2017; van Weeghel et al., 2019). Yet, whilst models such as CHIME reflect an increasingly positive, person-centred view of recovery in service delivery, service users have highlighted that many barriers still hinder progress towards personal recovery (Alyahya et al., 2022; Stuart et al., 2017; van Weeghel et al., 2019). This has led to the CHIME-D framework, which was proposed in a 2017 systematic review (Stuart et al., 2017), to include recognition of difficulties and challenges within the recovery process. This review, however, comprised limited exploration of potential barriers and it was acknowledged that there should be further examination of the perspectives of people with psychosis on these to inform the operationalisation of this user-based model of recovery (Stuart et al., 2017).

Against this background, qualitative explorations have identified social factors that can facilitate recovery, such as relationships and access to resources, but these too have emphasised the need for further exploration of the social aspects of recovery (Topor et al., 2011).

Crucially, to date research into lived experiences of recovery has disproportionately focused on male participants (Longenecker et al., 2010). This imbalance limits an understanding of how women experience psychosis and recovery, and risks reinforcing male-centred narratives in the literature. Whilst some studies have begun to explore gendered differences (Ayesa-Arriola et al., 2020; Carter et al., 2022), no research has yet thoroughly examined sex, gender and their potential intersections in shaping recovery experiences and possibilities. Recent literature reviews of lived experiences of psychosis (Alyahya et al., 2022; Wood & Alsawy, 2018) include women and identify potential barriers to recovery, but they too overlook the influence of sex and gender. Addressing this gap is important, as such insights could inform service provision and support recovery (Giordano et al., 2021; Haarmans et al., 2018; Riecher-Rössler et al., 2018).

Thus, to address this literature gap and begin to forge an evidence base for targeted interventions, this paper explores the following research question: What are the specific barriers to recovery from psychosis experienced by women and how do both sex and gender influence these experiences?

## Methods

### Study design

Qualitative secondary analysis (QSA) (Heaton, 2008) was undertaken of data collected for a study entitled *Women’s Lived Experiences of a First Episode of Psychosis: A Qualitative Analysis of the Influence of Gender on Day-to-Day Experiences   and Related Healthcare Needs (‘WEP’*; *PI: Anna Lavis*). WEP aimed to investigate the influence of gender on experiences of psychosis, including onset and course, healthcare access, and social and clinical needs.

Whilst the original WEP study centred on gendered experiences across the full course of psychosis, the focus on barriers to recovery was applied retrospectively in this secondary analysis, with the aim of developing novel insights into previously collected data (Heaton, 2008). This presents challenges, such as the potential mismatch between the original research questions and the new analytic focus (Heaton, 2008). To address this, we carefully re-examined the data and ensured the novel research question fit within the scope of the original interviews.

### Recruitment

Participants to WEP had taken part in Super EDEN (2010–2015), a large-scale, mixed methods study evaluating EIS for psychosis (*PI: Max Birchwood; Qualitative Leads: Helen Lester and Anna Lavis*). This study comprised 518 EIS service users.

For WEP, 41 participants were re-recruited from Super EDEN, initially via random sampling, with purposive sampling subsequently employed to ensure maximum socio-demographic variation (Palinkas et al., 2015). Thirty-one women and 10 men from Birmingham and Solihull, and Cornwall, were interviewed 2013–2015. All participants had experienced at least one episode of psychosis and been treated through EIS. Only the 31 women’s interview transcripts are included in this QSA to align with this secondary study’s aims. Table 1 provides a demographic summary. Most of the participants were White British (70.97%) and their ages ranged from 22 to 42 (*M* = 29, *SD* = 4.72).

**Table 1:** Participant demographics

Characteristics	Number of Participants		
	Number of Participants–Birmingham (*n*=18)	Number of Participants–Cornwall (*n*=13)	Total number of participants (*n*=31)
*Ethnicity*			
White British	9	13	22
Black British	2	0	2
Asian Pakistani	2	0	2
Mixed (Other Mixed Background)	2	0	2
Black Caribbean	1	0	1
British Muslim	1	0	1
White Other	1	0	1
*Service they were with at the point of interview*			
General Practice (GP)	9	9	18
Community Mental Health Team (CMHT)	4	9	13
*Employment status at the point of the interview*			
In Employment	6	7	13
Voluntary Work	1	0	1
Full Time Education	0	1	1
Unemployed	11	5	16
*Children at the point of interview*			
Yes	9	5	14
No	9	8	17
*Living status at the point of the interview*			
Living alone	5	1	6
Living with partner and /or children	11	6	17
Living with friends or family	2	6	8

### Data Collection

For WEP, participants took part in one in-depth semi-structured interview on their experiences of psychosis, from onset to the time of the interview. Interviews were conducted face to face in a location of each participant’s choosing. Some of the interviews were undertaken by the corresponding author, others by the study researchers. Audio recordings were transcribed verbatim by a professional transcription company.

Underpinned by the interpretive qualitative framework of medical anthropology (Lambert & McKevitt, 2002), which explores the social, structural and cultural aspects of a particular phenomenon, the interview topic guide was designed to allow participants to focus on the aspects of their lives surrounding a FEP they identified as most important.

### Reflexive Thematic Analysis

For this QSA, reflexive thematic analysis (RTA), following Braun and Clarke’s six step approach (Braun & Clarke, 2006, 2019), was chosen for its theoretical flexibility (Braun & Clarke, 2006, 2019) and its suitability for the heterogeneity of the dataset; participants were at different stages in their personal recovery and the topic guide had allowed them to focus on varied aspects of psychosis and recovery experiences, as they wished. Line by line coding was conducted as a primarily inductive process, to allow the process to remain data-driven. NVivo (QSR International Pty Ltd. Version 1.7.1, 2020), was used to organise the data and aid the coding.

It is recognised that multiple interpretations and meanings can be realised from the same dataset, depending on the researcher’s positioning (Braun & Clarke, 2022). Therefore, the analysis was conducted with consideration of credibility, transferability, dependability and confirmability (Lincoln & Guba, 1985), to ensure the methods were rigorous and findings insightful (Nowell et al., 2017). Acting as “auditable evidence” (Nowell et al., 2017), a reflexive journal was kept throughout analysis by the first author (Lock), detailing ideas, impressions and decisions. To minimise the effects of potential biases, pre-existing ideas and assumptions were also documented. Analytic triangulation, comprising independent coding, was conducted within the research team. Three researchers were involved in the process. Lock initially read and coded all 31 transcripts, whilst Lavis and Pendrous each independently coded two transcripts. The coding for these four transcripts was then compared to that from the dataset as a whole, as performed by the first author, and a continued process of discussion supported, assessed and refined the analysis as this progressed. This enabled an enriched analysis, with multiple interpretations considered (Braun & Clarke, 2013).

No prior theoretical frameworks were employed to conduct the analysis in accordance with the primary study’s epistemological framework of medical anthropology; nonetheless the findings have been explored in dialogue with relevant literature.

### Ethical Considerations

WEP received NHS ethical approval (13/WS/0226). Prior to the interviews, written informed consent was gained from each participant. Consent was verbally reconfirmed after each interview. The sponsor, Birmingham and Solihull Mental Health Foundation Trust, gave approval for this QSA.

### Findings

We identified four themes and 11 sub-themes relating to barriers to recovery from psychosis. The themes comprised individual factors: (1) Identity and (2) Temporality, and structural factors: (3) Social Imaginings of Psychosis and Stigma and (4) Socioeconomic Barriers. Larger quotations from participants' narratives are presented in blocks, with smaller ones denoted by italics within the text.

## Theme 1—Identity: Evolving Self-Perception and Diminished Self-Esteem

### Identity Conflict and a Longing for the Familiar Self

Many participants described how their sense of identity changed following their psychosis and continued to evolve long after the initial FEP. One woman, for example, described psychosis as *“completely life-changing”* and many reported that it took a long time to regain their sense of self following the resolution of psychotic symptoms, demonstrating the distinction between clinical and personal recovery. This was a long-term barrier to recovery, as participants feared they would never *“feel like me”* again, and thus would be unable to *“move on”* with life with or beyond psychosis:

I think once you’ve experienced psychosis, you can’t be the same person ever again. (White British Woman, aged 29)

Many participants reminisced about their lives prior to experiencing psychosis, describing their selfhood, abilities, and hobbies and highlighting what was missing or had been *“lost”:*

When I was eighteen that was the best of me. You know what I mean, I was young, having fun, going out to clubs... and now I don’t do those things no more. (Black Caribbean Woman, aged 28)

This sense of mourning or longing for previous lives and selves was sometimes accompanied by a tension in choosing an approach to recovery: attempt to revert to one’s ‘old’ identity or develop a new identity.

These struggles were, for some women, not only internal but patterned through gendered expectations, with participants feeling pressure to fulfil normative social roles such as being a mother or a partner, which in turn intensified identity loss. One participant described how the *“balancing act”* of maintaining multiple different sides of her previous identity whilst recovering left her feeling overwhelmed:

I lost my whole identity and it was just kind of like…I don’t know, one, getting my bond back with my son and be a good mum, two to re-find myself again, in a way, and try and get the old me back and then also, to try and be a wife at the same time […] it just felt like, I couldn’t be all those three people at the same time when I’m trying to get better. (White British Woman, aged 29)

### Loss of Trust in the Self Hindering Daily Life and Undermining Recovery

Many participants described feeling unable to trust themselves following a psychotic episode, making it difficult to undertake previously everyday activities like leaving the house, cooking, socialising, and personal care. This exacerbated some women's feelings of being unable to cope and reinforced the sense of not being, as some participants poignantly put it, *“normal”:*

You feel like everything gets on top of you and you’ve got all this responsibility, but you can’t trust yourself to carry out the tasks. It might be taking your medication, it might be not self-harming, whatever it is. But you can’t trust yourself, even with the best intentions. (White British Woman, aged 30)

Some participants described how psychosis had highlighted and exacerbated existing self-esteem issues that were both gendered and, often, also instigated by past relationships:

I think the fact that I was female did play quite a part in it, and I think the relationship side of things played a massive part in it. And erm, as a female, never feeling good enough and, self-esteem, I think for females, is a massive thing. (White British Woman, aged 29)

This lack of trust contributed to a loss of confidence in recovery as a realistically achievable goal:

I had no faith in myself, I had no belief in myself... I didn’t believe, honestly... didn’t believe that I could ever get any better, or do the things that I wanted to do, living with fear all the time. (White British Woman, aged 33)

Whilst some participants attributed low self-confidence to the experienced loss of identity, as discussed above, others linked it to the symptoms of psychosis itself. Positive symptoms such as paranoia and hallucinations had instigated self-criticism:

Examples would be things like, ‘Oh, you’re,’ erm, ‘You’ll never amount to anything,’ erm, ‘You’ll always fail,’ erm, ‘You’re ugly,’ whatever, whatever […]it had laid the foundation for leaving me feeling worthless. (White British Woman, aged 29)

### Body Image Disruption Being Overlooked in Clinical Care

Participants’ experiences of medication side effects meant that they sometimes struggled to adhere to their ongoing treatment plans long after the FEP. Most mentioned weight gain as an unfavourable consequence of anti-psychotic medication. These physical changes influenced women’s confidence and selfhood, acting as another manifestation of their fears of losing the *“old me”.*

I went to a size 14 to 16[…] that completely alienated me from everyone because I couldn’t wear any clothes to go anywhere. Nothing fitted me. It was awful. (White British Woman, aged 30)

I put on four stone within two months and that deeply, deeply, deeply affected me and made me massively depressed. […] A lot of men, it wouldn’t affect to a deep extent, as it did with females, I don’t think. (White British Woman, aged 24)

Despite its importance, the women described how their weight gain was underplayed and dismissed by clinicians and its negative impact on well-being underestimated. When one woman voiced her concerns to her care team, she was met with denial and felt that she had no other option but to stop her medication, potentially negatively affecting her recovery.

They did try to point out to me that it wasn’t causing my weight gain when I’d clearly gained about four clothes sizes and two stone […] they said ‘No, no, you must be eating, you’re gaining weight’ and I’m like ‘No, it’s because of the meds’ and they just, they weren’t prepared to accept that, so I stopped taking them. (White British Woman, aged 30)

## Theme 2—Temporality: How the Past and Present Impede Future Recovery

### Compounded Trauma—Past Experiences and the Impact of Negative Interactions with Mental Health Services

Several participants identified past traumatic experiences, such as having been abused as a child, sexual assault, and traumatic childbirth, as triggers for their FEP. Additionally, many women had been subjected to domestic violence and emotionally abusive relationships. These traumas contributed to the self-esteem issues discussed in **Theme 1** and manifested in the content of participants' psychotic symptoms. For example, one woman linked repressed memories of her past abusive relationship to her hallucinations and belief that all men wanted to harm her:

I just completely had this big meltdown and decided that everybody, if they were a man, had an axe under their coat and that they were chasing me round with axes. (White British Woman, aged 30)

These gendered traumatic experiences hindered recovery for many participants because it was only after having been supported to address them that they felt able to *“move on”* and focus on their future.

This process took a large emotional toll on the women and was described as not being adequately supported by healthcare services, which delayed the recovery process:

I don’t think they actually tried to target and, you know, help me with the underlining problems towards recovery. I think they were just trying to mask around it.

And

That was never really a topic of conversation, I suppose. They just wanted to, kind of, pussy foot around the outsides of things, really, I suppose, instead of going to the crux of the problem, and that only, you know, the male part of it, I suppose, only came out when I did psychology, when I wasn’t under the influence of the team at the time. (White British Woman, aged 24)

Receiving a diagnosis of psychosis was also described as a negative experience that itself impacted recovery. Many participants felt there had been a lack of both support and explanation during a very vulnerable time. For some women, healthcare professionals’ failure to correctly identify early symptoms had delayed diagnosis and access to services, and thereby the support needed to begin a recovery process:

He [GP] stuck me on antidepressants for ages, about 15 months, and they didn’t do a single thing. I kept going back and it was like, ‘Oh, no, no, you’re all right, you’ve just got postnatal depression.’ He wasn’t interested at all. […] From 21 to 32 nothing was picked up. Even though I went to see GPs and I was in hospital nothing was picked up. (White British Woman, aged 42)

Crucially, other participants felt that the reluctance of EIS healthcare professionals to use diagnostic labels in the early stages of a psychotic illness hindered their long-term recovery. They felt it had denied them the ability to fully understand their symptoms, making it difficult to accept a diagnosis when later given, especially during the transition from EIS to a Community Mental Health Team (CMHT where a formal diagnosis was employed to guide treatment:

I asked for a diagnosis within the first year of becoming poorly. And I read a leaflet, on the ward where I was an inpatient, and self-diagnosed, actually, quite spot on. Erm, but then, when I asked and outright said, ‘Do I have...?’ erm, I was, I was told, ‘No, you have depression with psychosis.’ And I later found out that was because they thought that I wouldn’t handle the fact of the label. […] But, actually, at the time, I wanted to know what it was so that I could call it what it was. (White British Woman, aged 29)

When I left the Early Intervention Team I was being treated for psychosis and then I turned up at [CMHT] for my very first interview with the psychiatrist and was told I’d got schizophrenia in a rather offhand way, and I think that’s why I’ve had such problems dealing with my diagnosis because it wasn’t explained to me that I might have had that before that appointment. (White British Woman, aged 33)

### The Cyclical Nature of Psychosis Eroding Faith in Recovery

Most participants referred to their episodes of psychosis and recovery as “*cyclical*” with “*ups and downs*” rather than as a linear trajectory:

When you’re becoming unwell, it can go up and down, up and down, and then it’ll spiral downwards. It’s not, like, ‘Oh, you’re going to get ill,’ and it gradually gets worse, it, sort of, fluctuates, erm, and you get mini episodes, leading up to a bigger episode. (Black British Woman, aged 23)

Barriers to recovery, then, changed over time. This could depend on alterations in a participant's life stage but also on more micro day-to-day social or physiological changes. For example, some linked this variability to hormonal changes within their menstrual cycle, which had the potential to negatively impact their mental state, making them vulnerable to relapse:

I couldn’t cope with the hormones as well as all the mental health; I could cope with one or the other, you know, reasonably well, but together they were too much. (White British Woman, aged 30)

This sense of changeability acted as an obstacle to recovery by making it feel temporary, elusive, or uncertain. In periods of good health, participants felt unable to trust that relapse would not occur, as it had done before:

You know the whole thing where erm you’re always an alcoholic and however long you’ve been recovered you could always go back to it, there is that kind of sense. (White Other Woman, aged 33)

### Stagnation Creating a Lack of Hope for the Future

Threaded through many of the transcripts was a sense of stagnation or pause; the women’s lives had been put on hold, often for years, whilst they struggled with psychosis. There were feelings of frustration and injustice that they had *“lost”* so much time to their illness. This was described as *“demoralising”,* and as reducing their motivation to recover:

I feel like my life’s been stolen from me. (White British Woman, aged 33)

I get really upset in that eight years I could have done so much. (Asian Pakistani Woman, aged 25)

Some participants recounted being unable to relate to old friendship groups, feeling *“left behind”* whilst their peers had *“moved on”* with education, work, and social opportunities. This was a barrier to recovery as their support networks, which are known facilitators for recovery, weakened:

All my friends now have got relationships, or they’ve got kids and they’ve got their own lives, they’re doing school stuff and kid stuff and family things, and I’m just here, like, nothing. (White British Woman, aged 32)

Them company ain’t there anymore, they’ve gone about their business[…]. Everyone’s moved on, everybody’s moved out. (Black Caribbean Woman, aged 28)

Participants reported feeling lonely and disheartened, which made the task of “*rebuilding*” their lives seem daunting, and at times, “*impossible*”. Many women experienced this hopelessness as a felt inability to visualise themselves recovering:

I don’t have the tools almost or I’m missing something I feel very empty, I feel empty, really empty. (White British Woman, aged 29)

There was a feeling of hopelessness, as well, thinking that I can’t do anything to cope. (Black British Woman, aged 23)

## Theme 3—Social Imaginings of Psychosis and Stigma: The Negative Impacts of Others’ Perceptions

### Stigma Causing a Loss of Support Systems

Whilst for some participants, family and friends had played key positive roles in their recovery, others had experienced stigma from close relations:

Friends who used to know me, they say hello, bye, but they don’t say anything other than that because I think they’re being careful, you know they’re kind of scared. (Asian Pakistani Woman, aged 25)

As a result, some women felt reluctant to discuss any ongoing mental health struggles with friends out of fear of their reactions or not wanting to *“burden”* them:

When you’ve got mental health issues, there’s a social, sort of, stigma, so you kind of feel isolated, and sometimes you feel like you can’t talk about it to your friends, because you’ll become estranged from them. (Black British Woman, aged 23)

Consequently, many participants lacked a support network and described feeling isolated, which was detrimental to the continued process of recovery.

### Healthcare Prejudice Leading to Reduced Engagement

Many participants described fearing that they would encounter prejudice if they sought healthcare treatment, either for psychosis or more widely:

I didn’t want to go [into mental health services]. And I think that was probably a lot of prejudice, thinking that if you went to a hospital [...] then you were mental if you went. (White British Woman, aged 30)

In addition, several participants reported feeling consistently *“not listened to”* by healthcare professionals who, they said, underestimated their ability to participate in the management of their own health, due to their history of psychosis:

Once somebody figures out that you’ve got a problem like that, that’s when they stop taking you seriously. (White British Woman, aged 33)

She [GP] just looked at me like I was completely like mental and talked to me like I was child, and I was like, don’t talk to me like that just because I’ve got mental problems doesn’t mean I’m stupid. (White British Woman, aged 26)

Additionally, there were many instances of diagnostic overshadowing recalled in general practice in particular; where participants’ physical complaints were attributed to their mental illness, despite being unrelated:

I’d started wheezing, and it was probably either something to do with smoking or quitting smoking around that time, but erm he [GP] didn’t examine my chest, he just said, ‘So, how’s the psychosis?’ (White Other Woman, aged 33)

These experiences damaged the women’s trust in healthcare professionals, reducing their willingness to engage with services. This hindered recovery by delaying access to further support, if needed in times of declining mental health, as well as exacerbating feelings of stagnation, noted above, with their identity externally defined solely through psychosis.

### Media Depictions Perpetuating False Stereotypes

Although participants acknowledged increasing media representation of mental illness as positive, they felt the media presented an inaccurate depiction of living with mental health challenges. Many referred to sensationalised plots on television, which generated stigma through fearmongering:

I think it’s got people talking about it, which isn’t necessarily a bad thing. But I think it’s very interesting that on the programmes, the most exciting bit that happens in the programme is, ‘Oh, the alarms have been pulled. Oh, people are running off to hold somebody down who’s trying to hurt themselves, or hurt somebody else’, or, and it’s all this big hoo-ha. And actually, there’s very little on recovery; very little on, on a carer’s perspective; very little on, ‘Well, actually, what is that person going through?’ and looking through compassionate eyes. (White British Woman, aged 29)

The participants described such media depictions as limiting their potential for progression in many areas: finding employment, forming social groups, and accessing healthcare, thus impacting their ability to re-join society and achieve social recovery.

## Theme 4—Socioeconomic Barriers: The Hopelessness of Inequality

### Poverty and Inadequate Housing

Almost all participants mentioned the financial burden associated with mental illness, with many being unable to work during and after a FEP. Some expressed shame and low self-esteem that they related to a continued need for benefit (welfare) payments long after their acute illness:

I’m ashamed of the fact that I can’t work full time and that I’ve had to claim out of work benefits. (White British Woman, aged 32)

Complicated administrative processes had also prevented participants from accessing adequate financial aid. Some described pressure to *“jump through hoops”* to prove their need, causing distress and contributing to feeling a lack of agency over their lives:

The whole benefits thing has pissed me off really bad that’s made me ill, very ill and for a long time I wouldn’t even pursue it even though it’s stupid because you’re basically hanging yourself because you need the money. (White British Woman, aged 29)

Recovery was also hindered by some participants’ living environment, including inadequate housing that was crowded and/or dilapidated and posed a hazard to physical and mental health:

All I want, is somewhere to live. I mean if I was sick now and I can’t get anything, that means I can’t get nothing when I’m better, with damps on the walls.[...] There’s no gas in the flat, it’s just electric and it’s cold in the flat. No matter how warm it is outside, inside’s always cold. (Black Caribbean Woman, aged 28)

The lack of a safe, habitable and comfortable space in which to live had significant negative impact on participants’ emotional well-being, leading them to feel unsettled, unsafe and distressed, thereby unable to focus on recovery or the future more broadly.

Many participants also described struggling with the presence of large debts that had accrued over the course of their illness, which meant they were unable to escape this inadequate housing or a future of ongoing financial precarity.

### Stifled Opportunity—Unemployment After Psychosis

Difficulties finding or maintaining employment were commonly reported by the participants as enduring long after the acute illness. Many factors that prevented the women from attaining employment were described, such as continuing symptoms being incompatible with a workplace environment and a lack of confidence:

They’re telling me stuff like you can become whatever you want. Apprenticeships out there, this out there. But I’m thinking hang on, how can I do that if I can’t mix with people? You know? And erm, if I can’t mix with people, how am I gonna get my job? (Asian Pakistani Woman, aged 24)

Some participants reported experiencing stigma during job interviews, such as potential employers asking intrusive questions that made them feel uncomfortable and othered. This reduced their confidence in their ability to reintegrate into society and achieve recovery:

The way they interviewed me was unacceptable, the way they was asking me about my, erm, illness and, erm, ‘When do you hear voices?’ and all, it was quite intimidating. (Black Caribbean Woman, aged 29)

Unemployment was described as a significant barrier to recovery by participants; many missed the routine of going to work in  providing structure and motivation to leave the house, which was described as important for maintaining mental well-being:

Well, I used to have structure and routine through work and, you know, the little pleasures like getting home from a hard day and relaxing and being happy…I don’t have that feeling anymore; I don’t have the buzz of wage—payday, you know, and I don’t get the social side of it. So, work’s been a big thing. (White British Woman, aged 33)

A notion of *“fitting in”* to society as key to recovery resonated across the transcripts. It was described by participants as allowing them to feel *“normal”*, thereby reducing isolation or alienation caused by stigma, as described in **Theme 3**. Unemployment then, was a significant social barrier. It also substantially contributed to the sense of entrapment posed by ongoing debt. As such, finances, housing, and unemployment were significant structural barriers to recovery.

## Discussion

This paper has extended understandings of women’s experiences of psychosis by exploring barriers to recovery and their intersections with both sex and gender. Whilst participants were recruited based on female biological sex, the focus of this QSA was lived experience and, as such, gender roles and expectations were also analysed and will be discussed.

### Identity: Loss in Recovery, Recovery as Loss

Across the participants’ interviews there was a pervasive sense of loss. Whilst a characteristic feature of psychosis may clinically be framed as a loss of touch with reality (Stanghellini & Ballerini, 2002), this research also highlighted loss of friends and social groups; independence, financial means and stability due to structural barriers; and of hope. Loss of personal identity emerged as a particularly significant barrier to recovery.

The significance of personal losses linked to psychosis has been previously highlighted in relation to recovery (Fusar-Poli et al., 2022). But, our research has extended this by showing the central significance of loss of trust in oneself and one’s ability to recover. This self-doubt has been attributed in the existing literature to the nature of psychotic symptoms, such as criticising auditory hallucinations (Ritunnano et al., 2022). However, our findings challenge this claim of self-doubt as purely symptom driven and therefore as ensuing outwards from within a space of illness. Instead, participants’ narratives demonstrated these changes to their sense of, and trust in, themselves to be socially instigated and patterned through the effects of stigma and societal projections.

In tandem, qualitative research has described the complexities of the relationship between psychosis and changing identity (Boydell et al., 2010; Harris et al., 2022; Jenkins, 2015). Importantly, in this study participants’ struggles with identity were not solely attributable to psychotic symptoms or internal processes of self-doubt, but rather were shaped by social pressures and gendered cultural expectations. Several women expressed distress at not being able to fulfil normative roles such as being a mother, maintaining stable relationships, or contributing as a caregiver. That all of these are culturally framed as markers of a “proper” female identity (Adams, 2014), may come to take on even greater significance for women as they tend to develop psychosis later in life (Giordano et al., 2021) when they are more likely to have dependents. The weight of these gendered ideals compounded the loss of self-experienced in psychosis, positioning recovery not only as regaining health but also as navigating the social consequences of failing to embody expected feminine roles.

Barriers to recovery, then, do not arise from individual deficits but are, rather, forged by gendered expectations, for example around appearance, caregiving and normative life trajectories. In fact, recovery itself can intensify such pressures, as participants described seeking to return to normative roles but being reminded of *"biological clocks"* and expectations of relationship formation and family planning.

### Temporality and Differing Meanings of Recovery

Participants’ narratives of disrupted temporality reflect more than the disorienting effects of psychosis itself. Concerns about *“lost time”* were deeply tied to culturally scripted timelines for life events. The urgency to catch up on milestones heightened anxieties about recovery, creating pressure to return quickly to social functioning, which again positioned recovery within gendered cultural norms.

Both survivor voices and qualitative research have confirmed that personal recovery in psychosis is not merely—or perhaps not at all—the absence of clinical symptoms (Bellack, 2006; Pitt et al., 2007), that it is cyclical rather than being defined by a fixed end goal (Bellack, 2006; Leamy et al., 2011), and may be fraught with ambivalence and negotiated dilemmas (Jenkins & Carpenter-Song, 2005, 2008). Yet, much previous research has attempted to define normative time frames for recovery, ranging vastly, from one to five years of the so-called ‘normal’ functioning (Bellack, 2006).

Our findings challenge such definitions and demonstrate that many barriers to recovery, such as low self-esteem, stigma, and unemployment, continue to permeate women’s lives, and continue to be imposed on them by society, long after clinical symptoms subside.

Recovery was conceptualised by our participants as the process of overcoming these long-enduring social and structural barriers. These findings thereby raise questions around what can be considered ‘fully recovered’, what constitutes ‘normal’ functioning, and who can and should determine this, as well as how recovery can be contextually supported rather than hindered.

Despite core commonalities across the transcripts, participants had varied priorities for, and concerns regarding, recovery, each conceptualising it in different ways. Defining it, moreover, proved challenging for the participants, elucidating a felt tension between wishing to revert to a previous identity or forging a new one. On the one hand, this supports the call for recovery to be recognised as a self-defined concept (Law et al., 2012). On the other, it also demonstrates that, whilst attempts to characterise recovery may provide valuable guidance for clinicians and support systems aiming to deliver recovery-oriented care, they also risk oversimplifying the changeability and longevity of the recovery process, as well as its social embeddedness. Our findings therefore evoke critiques, especially by survivor academics, of the increased individualisation of recovery that has accompanied its “mainstreaming”. Moving away from the social justice perspectives of the survivor movement, this mainstreaming has been argued to dislocate the individual with psychosis from the social relations and contexts that shape such experiences and also to enact a normalising discourse (Rose, 2014).

### Healthcare Service Failings: Diagnosis

As well as loss of trust in oneself, qualitative research has described how delusions and paranoia in FEP can create a loss of trust in others, including healthcare professionals (Fusar-Poli et al., 2022). Our research found that a loss of trust in clinicians can also lead to reduced help-seeking, creating a barrier to recovery. Crucially, healthcare professionals and policies can contribute to and worsen these trust issues, such as through a lack of transparency and clarity, particularly around diagnosis (Farooq et al., 2019). Clinicians may often hold back a diagnostic label to protect service users from the “harms of stigma” (Farooq et al., 2019), but our findings suggest this approach can have mixed effects.

The presence of these paternalistic notions in our dataset may be related to gender, underpinned by a societal perception of women as emotional and vulnerable (Eagly & Koenig, 2006). However, it also reflects EIS’ gold standard to use the term “First Episode Psychosis” (FEP) rather than giving a definitive diagnosis (National Institute for Health and Care Excellence, 2016). Part of the rationale for this is to allow service users to address their symptoms without having to suffer the potential stigma of a diagnostic label (National Institute for Health and Care Excellence, 2016). This aligns with survivor and activist critiques, which argue that diagnostic labels can be pathologising, dehumanising, and constraining (Timimi, 2014). Indeed, this was evidenced in our findings as the women experienced stigma due to their diagnosis, such as when attending job interviews.

However, whilst the term FEP may be beneficial in that it recognises the possibility of diagnostic uncertainty (National Institute for Health and Care Excellence, 2016), our participants highlighted an unintended consequence of the EIS approach: reduced clarity about their own diagnosis. This illustrates the paradoxical role of diagnosis: it can provide understanding, validation, and treatment guidance, supporting recovery when used thoughtfully, but it can also reinforce stigma and limit social participation. In this way, diagnosis is socially mediated, shaped not only by clinical use but also by broader social, structural, and gendered contexts.

### Healthcare Service Failings: The Need to Address Trauma

This study found that a key shortcoming of mental health services is a failure to recognise the significance of past trauma both to women’s experiences of psychosis *and* as a barrier to recovery. The failure to provide a space to discuss and explore trauma within UK Mental Health Services emerged as a significant long-term barrier to recovery for women.

The link between experiences of trauma, particularly adverse childhood events, and psychosis is well recognised (McManus al., 2016; Fisher et al., 2009; Varese et al., 2012) and trauma is discussed within many qualitative accounts of psychosis (Campodonico et al., 2022; Nixon et al., 2010). Although both men and women can be affected by trauma and abuse, gender may play a role in how people with psychosis respond to childhood abuse. Fisher et al. (2009) suggested that women are more likely to internalise these experiences, leading to paranoid delusions and suspiciousness of others. The nature of the reported abuse is also gendered, as women are more likely to experience sexual abuse, rape, and domestic abuse (Fisher et al., 2009). Our results reflect this social patterning and show the range of traumatic experiences that women face, particularly, at the hands of men, for example, ex-partners. Our participants, many of whom described experiences of trauma, discussed the multi-faceted impact this had on them, reducing their confidence, and contributing to struggles of identity, as well as manifesting in the content of their delusions and hallucinations.

“Exploration of prior trauma” is now included in EIS’s policy (Royal College of Psychiatrists, 2018). However, given that these interviews were conducted in 2013–2015 and NICE's Schizophrenia Management Guidelines were updated in 2014 (National Institute for Health and Care Excellence, 2014), these changes may not have yet taken effect when the interviews were conducted. These policy changes represent a move towards Trauma-Informed Care (TIC) within the NHS (see Emsley et al. 2022). Current literature assessing the extent to which these policies are implemented is inconclusive: a review of CMHT case notes found inconsistencies that suggest trauma enquiry is still not part of routine practice (Neill & Read, 2022). However a 2022 study, which interviewed clinical psychologists working for EIS, concluded that they were conducting more trauma-informed practice (Mountjoy et al., 2022). Despite this, there was no mention of trauma-specific therapies being implemented and no discussion of the needs of women specifically. That CMHTs may still be lacking in this area perhaps exposes a disconnect between the two services. Whilst service users should not be pressured to undergo trauma-focused therapies, our findings align with that of Martin et al. (2023) in arguing that this kind of support should be offered.

### Social Imaginings of Psychosis and Lack of Agency

Finally, the structural barriers to recovery highlighted by our participants convey the lack of agency they experienced over their own lives and/in recovery. Almost all participants had experienced stigma; being referred to as *“freak”, “crazy”,* or *“mad”.* These findings echo those of existing qualitative research; participants described feeling *“weird” *and like they *“stand out”* (Burke et al., 2015). External stigma became internalised, resulting in critical internal voices during psychosis, which then contributed to their enduring struggles with identity and the sense of not feeling *“normal.”* Crucially, the impact of stigma continued long after the FEP, thus presenting a long-term barrier to recovery.

A key finding was that weight gain acts as a significant stigma and thus barrier to recovery. Stigma was compounded by feeling that one’s physical appearance did not conform to gendered societal norms. As a side effect of medication, this altered appearance became yet another aspect of life over which participants felt they had no agency. The issue was closely tied to both sex and gender: biologically, women are more likely to experience significant weight gain from anti-psychotic medication (Seeman, 2012), whilst socially, participants reported greater pressure than men to maintain a *“well”* presentation which they described as undermined by weight gain.

Ethnographic research by Jenkins and Carpenter-Song (2005) further illuminates this experience through the “crazy or fat” dilemma, in which individuals feel they must choose between the negative consequences of medication, most prominently weight gain (“fat”), and the potential worsening of their condition if they do not take it (“crazy”). This framing captures how stigma is not only external but also internalised in profoundly gendered ways. For women in particular, weight gain may contribute to a sense of being “genderless”, stripping them of identities that may be tied to normative Euro-American ideals of femininity which centre thinness (Swami et al., 2010). Our findings echo this, showing that weight gain not only affected participants’ sense of self but also constrained their agency and hope, reinforcing the social and gendered dimensions of stigma and compounding the barriers to recovery.

In addition, experiencing negative medication side effects, such as weight gain, was one way in which our participants felt their attempts to be hopeful were thwarted by a lack of agency. The notion of hope has garnered attention in the existing literature (Green & García-Mieres, 2022) as a key aspect of recovery. However, our findings show how hope needs to be recognised as constrained and curtailed by social context and even structural violence (Farmer, 1996).

It is only within the last decade that structural barriers have been acknowledged and reflected in UK Government mental health policy. For example, strategies such as The Individual Placement and Support Model, which aims to support individuals with a mental illness to gain competitive employment (Department of Work & Pensions and Department of Health, 2017; Slade et al., 2014), have been implemented. It has also been recognised that inequality, poverty, sexism, racism, and other social adversities are factors in the development of mental illness (Belle, 1990; Monte et al., 2008).

However, this study goes further by highlighting that structural factors can also significantly impede recovery. For the women in this study, the social contexts in which both their psychosis and recovery were experienced fostered a pervasive sense of a lack of agency, undermining their attempts to generate and maintain hope. This echoes Myers’ (2016) analysis of the need for - and conditions that forge resources for - moral agency in recovery from severe mental illness. Moral agency as defined by Myers is the ability to lead a “good life,” including the pursuit and achievement of personal goals and plans (Myers, 2016). Importantly, moral agency is exercised within gendered social contexts. For women, this may often entail navigating societal expectations regarding caregiving, relational responsibilities, and acceptable behaviour, meaning that their attempts at moral agency are constrained by both structural and gendered pressures.

In our study, the structural barriers of unemployment, poverty and financial instability, and inadequate housing were frequently discussed as instigating a sense of defeat. Participants also reported receiving insufficient practical support from health and social care services, further curtailing hope. This highlights the necessity of understanding women’s experiences of psychosis and recovery not only in terms of structural disadvantage but also in relation to the gendered conditions that shape moral agency in an unequal world.

## Study Strengths and Limitations

QSA allows maximisation of the utility of data from transcripts (Heaton, 2008), facilitating the exploration of new themes. With QSA, however, there is a risk of a lack of familiarity with the dataset as the author who led the analysis (Lock) had not conducted the interviews. This was countered by allocating sufficient time for phase one of Braun and Clarke’s thematic analysis: familiarisation, to allow full immersion in the data and by the corresponding author (Lavis) having written the topic guide and conducted some of the interviews.

Secondly, although the interview topic guide included questions on recovery, there were no specific questions addressing *barriers* to recovery, hence it was possible that potential barriers were missed. However, due to the open anthropological approach of the study, a reflexive space was afforded in the interview to each topic that participants chose to focus on, as the interviewers followed participants’ conversational threads. Therefore, where recovery was covered, participants discussed this in ways that highlighted what was important to them, which elucidated nuances around barriers and facilitators.

Thirdly, most of the participants identified as White British. This lack of ethnic diversity is a limitation, particularly as the experiences of people from minority ethnic backgrounds are underrepresented in the literature on both psychosis and recovery (Redwood & Gill, 2013). Our analysis was not stratified by ethnicity, and although the themes generated were shared across participants' narratives, future research would be strengthened by examining this important intersection in greater depth. Despite this limitation, the varying social circumstances of the participants and heterogeneity of their life experiences gave rise to a rich dataset and the sample size of 31 is larger than average when compared to studies of similar methodology (Braun & Clarke, 2021).

Additionally, this study did not systematically examine the relative contributions of medication, psychotherapy, and other forms of clinical and social support during and following FEP. Future research would benefit from exploring how these different forms of intervention interact with gendered experiences of psychosis and recovery.

Finally, the participants’ accounts were retrospective, as they were interviewed at varying time spans following the FEP. Whilst the analysis may be subject recall bias, this timing may also have offered the opportunity for deeper reflection, with participants being better able to identify aspects of their experience that acted as barriers to recovery over time.

## Conclusion

This study has highlighted the complex interplay of individual and structural barriers that shape women’s recovery following psychosis. Individual barriers include changes to personal identity, diminished self-esteem, feelings of lost time and interrupted life trajectories, and the enduring impact of past trauma. Structural barriers are stigma—which is often gendered, poverty, inadequate housing, unemployment, and insufficient support from health and social care services. These collectively constrain women’s agency and sense of hope. Notably, many individual challenges are compounded by structural factors, particularly the failure of services to recognise that unresolved past traumas not only contribute to the development of psychosis but also persist as barriers to recovery. Our findings demonstrate that both sex and gender are crucial considerations to understanding these barriers: biological differences influence symptom experiences and treatment effects, whilst gendered, social expectations shape recovery trajectories and internalised stigma.

By foregrounding the lived experiences of women this study has extended the existing literature by showing how recovery is deeply embedded in social contexts. These insights underscore the need for a more personalised approach to treatment and support for women; however, due to the pervasive nature of structural barriers, population-based interventions and societal shifts are also necessary. We recommend that further research examines sex- and gender-specific interventions in this context, with a future view to integrating these into clinical practice, to ensure recovery support for women is both inclusive and effective.

## Declarations

### Disclaimer

This paper/report presents independent research funded by the National Institute for Health Research (NIHR). The views expressed are those of the author(s) and not necessarily those of the NHS, the NIHR or the Department of Health and Social Care.
